# A Hybrid Multimodal Cancer Diagnostic Framework Integrating Deep Learning of Histopathology and Whispering Gallery Mode Optical Sensors

**DOI:** 10.3390/diagnostics16060848

**Published:** 2026-03-12

**Authors:** Shereen Afifi, Amir R. Ali, Nada Haytham Abdelbasset, Youssef Poulis, Yasmin Yousry, Mohamed Zinal, Hatem S. Abdullah, Miral Y. Selim, Mohamed Hamed

**Affiliations:** 1 Computer Science and Engineering Department, Faculty of Media Engineering and Technology (MET), German University in Cairo (GUC), New Cairo 11835, Egypt; nada.abdelaziz@student.guc.edu.eg (N.H.A.); youssef.poulis@guc.edu.eg (Y.P.); 2 Mechatronics Engineering Department, Faculty of Engineering and Materials Science (EMS), German University in Cairo (GUC), New Cairo 11835, Egypt; mzinal2015@gmail.com (M.Z.); htmsamir@gmail.com (H.S.A.); miraltawfiiq392@gmail.com (M.Y.S.); 3 ARAtronics Laboratory, Mechatronics Engineering Department (MCTR), German University in Cairo (GUC), New Cairo 11835, Egypt; 4 Product Design, Faculty of Applied Sciences and Arts (FASA), German University in Cairo (GUC), New Cairo 11835, Egypt; yasmin.yousry@hotmail.com; 5 Rostock University Medical Center, 18057 Rostock, Germany

**Keywords:** cancer detection, histopathology, WGM optical sensors, deep learning, EfficientNet, vision transformer, InceptionV3, DCGAN, data augmentation, multi-class classification

## Abstract

**Background/Objectives:** Biopsy examination remains the gold standard for cancer diagnosis, relying on histopathological assessment of tissue samples to identify malignant changes. However, manual interpretation of histopathological slides is time-consuming, subjective, and susceptible to inter-observer variability. The digitization of histopathological images enables automated analysis and offers opportunities to support clinicians with more consistent and objective diagnostic tools. This study aims to enhance cancer diagnosis by proposing a hybrid framework that integrates deep-learning-based histopathological image analysis with Whispering Gallery Mode (WGM) optical sensing for complementary tissue characterization. **Methods:** The proposed framework combines automated tumor classification from histopathological images with biochemical signal analysis obtained from WGM optical sensors. Deep learning models, including EfficientNet-B0, InceptionV3, and Vision Transformer (ViT), were employed for binary and multi-class tumor classification using the BreakHis dataset. To address class imbalance, a Deep Convolutional Generative Adversarial Network (DCGAN) was utilized to generate synthetic histopathological images alongside conventional data augmentation techniques. In parallel, WGM optical sensors were incorporated to capture subtle tissue-specific signatures, with machine learning algorithms enabling automated feature extraction and classification of the acquired signals. **Results:** In multi-class classification, InceptionV3 combined with DCGAN-based augmentation achieved an accuracy of 94.45%, while binary classification reached 96.49%. Fine-tuned Vision Transformer models achieved a higher classification accuracy of 98% on the BreakHis dataset. The integration of WGM optical sensing provided additional biochemical information, offering complementary insights to image-based analysis and supporting more robust diagnostic decision-making. **Conclusions:** The proposed hybrid framework demonstrates the potential of combining deep-learning-based histopathological image analysis with WGM optical sensing to improve the accuracy and reliability of cancer classification. By integrating morphological and biochemical information, the framework offers a promising approach for enhanced, objective, and supportive cancer diagnostic systems.

## 1. Introduction

Early and accurate diagnosis of cancer is essential to improve treatment outcomes and reduce mortality. Histopathological examination of biopsy samples remains the clinical gold standard for cancer detection, but manual assessment is time-consuming, subjective, and prone to inter-observer variability [1,2,3,4]. These limitations can delay timely decisions and affect diagnostic consistency, motivating the development of automated and more reliable methods for tumor classification.

Recent advances in artificial intelligence (AI) have enabled the use of deep learning (DL) models for histopathological image analysis [5,6,7,8,9,10,11,12]. Convolutional neural networks (CNNs) and attention-based architectures such as Vision Transformers (ViT) have shown strong performance in both binary and multi-class tumor classification tasks [13,14,15,16,17,18,19,20,21,22,23,24]. To address challenges such as limited annotated datasets and class imbalance, generative models like Deep Convolutional Generative Adversarial Networks (DCGANs) have been used to augment data, improving model generalization and robustness. Segmentation models (e.g., SegNet, U-Net) and transfer learning approaches have further enhanced the capability of DL frameworks to capture subtle morphological patterns in histopathological images [25,26,27,28,29].

Building upon these developments, histopathological image analysis using deep learning has emerged as a powerful tool for automated tumor classification [8,10,14,18,19,20]. Segmentation models such as SegNet and U-Net have been applied with varying success depending on dataset balance and noise levels [26,28], while transfer learning approaches, including InceptionV3 [5] and AlexNet [6], have achieved high accuracy but face generalization challenges due to limited data diversity [9,13,27,29]. Despite high reported performance, for example, up to 99.89% in binary classification using Vision Transformer (ViT) [7], many studies remain constrained by dataset bias, model scalability, and the lack of multimodal integration [8,9,18].

In addition to deep-learning-based approaches, probabilistic models such as Bayesian Networks have been explored in medical decision-support systems due to their ability to model uncertainty, represent causal relationships, and handle complex, multidimensional clinical data. Bayesian Networks offer an interpretable framework in which dependencies between variables can be explicitly defined, making them particularly appealing in clinical contexts where transparency and explainability are essential. However, their applicability to high-dimensional data such as histopathological images is often limited by the need for carefully designed probabilistic structures and expert-defined priors. As a result, recent research has increasingly favored deep learning models for image-driven cancer diagnosis, while probabilistic methods are more commonly used as complementary or decision-level tools in multimodal diagnostic systems [30,31,32,33].

While deep learning significantly enhances histopathological analysis, complementary diagnostic modalities can provide additional, high-sensitivity information to further improve early detection and decision-making. Among these, Whispering Gallery Mode (WGM) optical sensors have emerged as a promising platform for label-free chemical and biomedical detection [34], offering particular potential for early-stage cancer diagnosis. Whispering Gallery Mode (WGM) micro-resonators are optical microcavities, such as microspheres, microtoroids, or microrings, that tightly confine light via total internal reflection along curved surfaces, achieving extremely high quality (Q) factors and small mode volumes, and enabling sensitive, high-speed detection when coupled with tunable laser interrogation and rapid spectral tracking techniques [35]. The sensing mechanism relies on the evanescent field, which extends slightly beyond the resonator boundary into the surrounding medium, allowing detection of subtle environmental changes such as solvent diffusion or molecular interactions [36]. When target analytes, including proteins, viruses, or chemical molecules, interact with or bind to the micro-resonator surface, they induce minute perturbations in the local effective refractive index, producing measurable shifts in the WGM resonance wavelength that can be tracked to monitor molecular-level interactions with high sensitivity [37]. Binding events may also introduce absorption or scattering losses, reducing the resonator’s Q-factor and potentially affecting the accuracy and sensitivity of WGM shift measurements [38]. WGM sensors are highly attractive due to their ultra-high sensitivity, compact size, and label-free detection capabilities, which are essential for early disease diagnosis and precise biochemical monitoring. Their extreme responsiveness to nanoscale refractive-index variations enables the detection of individual particles, as demonstrated using polymeric WGM micro-optical sensors for monitoring chemical impurities in water [39].

Artificial neural networks (ANNs) can serve as digital twins, reproducing WGM outputs with comparable accuracy while enhancing robustness. Experiments with the GUCnoid 1.0 humanoid robot demonstrate that ANN predictions closely match actual WGM measurements, highlighting the potential of machine-learning-assisted WGM sensing for both biomedical and engineering applications [40]. This principle can be extended to single-cell detection in biomedical applications. Integration with microfluidic platforms further enhances the system by enabling precise sample manipulation, targeted surface functionalization, and multiplexed sensing within compact lab-on-a-chip devices. Furthermore, applying machine learning to WGM sensor outputs allows automated extraction and classification of biochemical signatures, enhancing the accuracy and efficiency of single-cell analysis.

Building on these technological advancements, this study proposes a hybrid cancer diagnostic framework that integrates deep-learning-based histopathological image analysis (Pipeline A) with machine-learning-enabled WGM optical sensing (Pipeline B). Pipeline A employs state-of-the-art CNNs, ViT models, and generative augmentation to improve tumor classification from biopsy images and constitutes the primary focus of the current work. Pipeline B is presented as a future-ready sensing platform, with its sensor architecture, microfluidic–optical interface, and experimental setup described in the manuscript, while machine learning is introduced conceptually as a tool for automated extraction and classification of WGM spectral signatures. Together, these two pipelines form a unified multimodal diagnostic system designed to enhance the accuracy, robustness, and clinical utility of cancer classification, while clearly delineating the current focus on Pipeline A and the future development of Pipeline B.

## 2. Materials and Methods

This section presents the proposed hybrid multimodal diagnostic framework, which integrates artificial intelligence-based histopathological image analysis with optical Whispering Gallery Mode (WGM) sensing to achieve cancer characterization. The overall objective is to combine morphological features extracted from tissue images with molecular-level optical signatures to improve diagnostic reliability, accuracy, and robustness.

**Figure 1:**
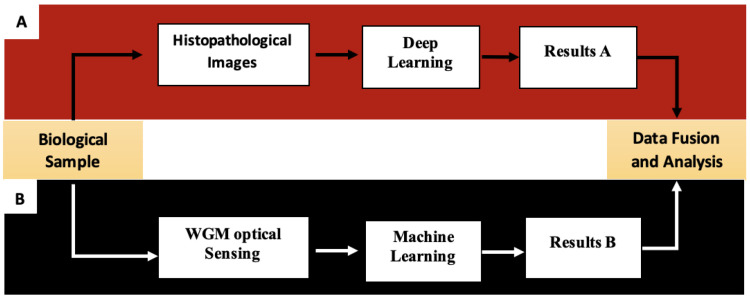
Hybrid diagnostic pipeline integrating histopathological image analysis and WGM sensing. The figure presents two parallel analytical pipelines derived from the same biological sample: Pipeline (**A**) processes histopathological images using deep learning to generate Result A, while Pipeline (**B**) employs Whispering Gallery Mode (WGM) optical sensing combined with machine learning to produce Result B. Both outputs are subsequently integrated in a unified Data Fusion and Analysis step to yield a comprehensive diagnostic assessment.

Building on this platform, the conceptual workflow of the proposed system is illustrated in Figure 1. A single biological sample is routed into two distinct yet complementary analytical pathways. In pipeline A shown in Figure 1, the sample is processed to generate histopathological images, which are subsequently analyzed by trained deep learning algorithms such as InceptionV3 or Vision Transformer (ViT) to produce a morphology-based diagnosis, referred to as Results (A). In Pipeline B, as shown in Figure 1, the same biological sample undergoes WGM optical sensing, leveraging the high Q-factor resonance and evanescent-field sensitivity of micro-optical resonators to detect biochemical markers at low concentrations. Machine learning is then applied to the resulting spectral data to automatically extract, classify, and interpret biochemical signatures, generating a parallel molecular dataset referred to as Results (B).

In the final stage, the proposed hybrid diagnostic framework employs a late (decision-level) fusion strategy, in which the two analytical pipelines operate independently. Pipeline A generates morphology-based predictions from histopathological images using deep learning models (Results A), while Pipeline B extracts biochemical signatures from Whispering Gallery Mode (WGM) optical sensing data through machine learning techniques (Results B). The outputs from both pipelines are then integrated within a fusion module to produce a unified multimodal diagnostic decision. This approach combines independent predictions rather than raw input data or intermediate feature representations, enhancing modularity and interpretability. At this stage, no attention-based fusion mechanism is applied; advanced strategies, such as attention-driven or weighted fusion, will be explored in future work.

### 2.1. Pipeline A: Histopathological Image Analysis and Deep Learning Framework

**Figure 2:**
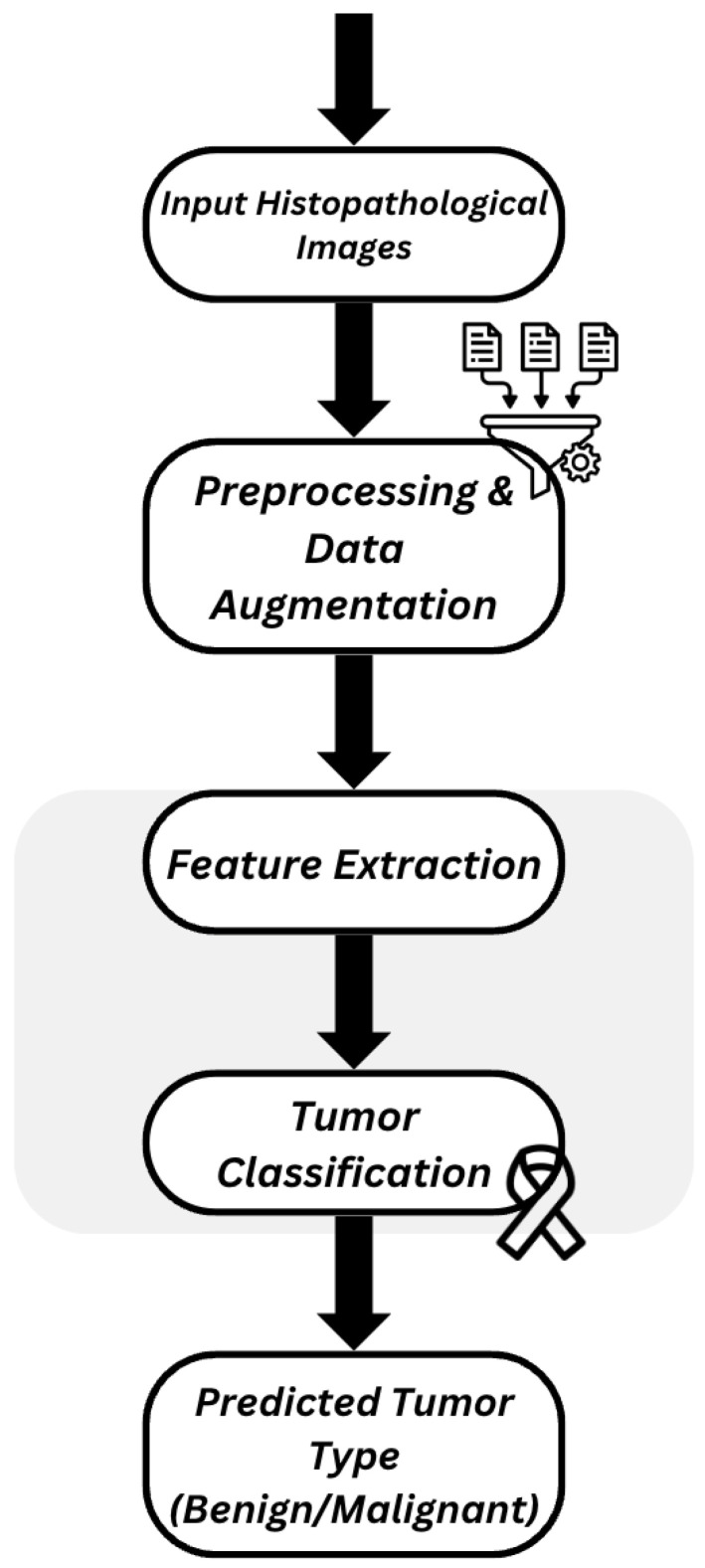
Proposed Classification system architecture.

This subsection describes the implementation and development of Pipeline A within the proposed hybrid framework. Figure 2 illustrates the architecture of the proposed histopathological image diagnosis system, which encompasses the datasets, preprocessing procedures, data augmentation strategies, and deep learning models used to generate the classification results.

#### 2.1.1. Dataset Selection

This study utilizes three widely recognized histopathological image datasets, BreakHis [41], BACH [42], and LC25000 [43], to ensure a comprehensive evaluation across multiple cancer types, magnification levels, and classification tasks.

**Figure 3:**
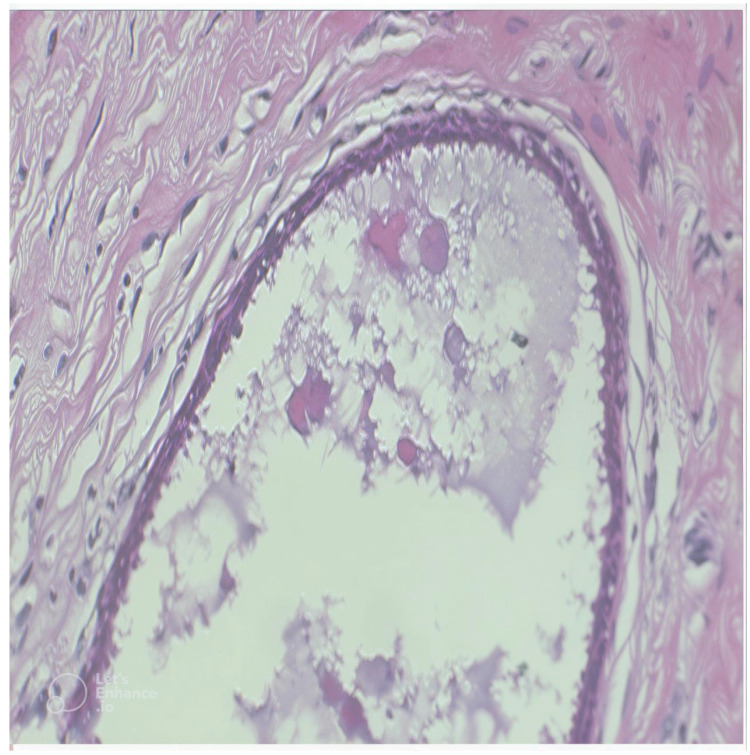
Sample of BreakHis dataset [41].

BreakHis is a benchmark dataset for breast cancer histopathology. It comprises 7909 microscopic images of breast tumor tissues captured at four magnification factors: 40×, 100×, 200×, and 400×. The dataset supports both binary classification (benign vs. malignant) and multi-class classification across eight subtypes (four benign and four malignant). All images are labeled at the image level, although no segmentation masks are provided, and a sample is shown in Figure 3. In this study, we focus primarily on the 40× subset to ensure consistency in magnification and fair comparison between models.

**Figure 4:**
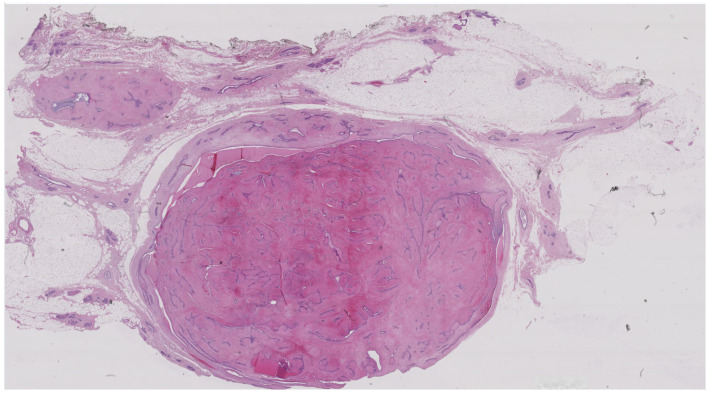
Sample of BACH Dataset [42].

BACH provides high-resolution histopathological images, including both cropped image patches and full whole-slide images (WSIs), as shown in Figure 4. The dataset is categorized into four histological classes: normal, benign, in situ carcinoma, and invasive carcinoma. BACH includes region-level annotations, allowing for both classification and segmentation tasks. Its WSI format offers a realistic evaluation scenario resembling clinical diagnostics.

**Figure 5:**
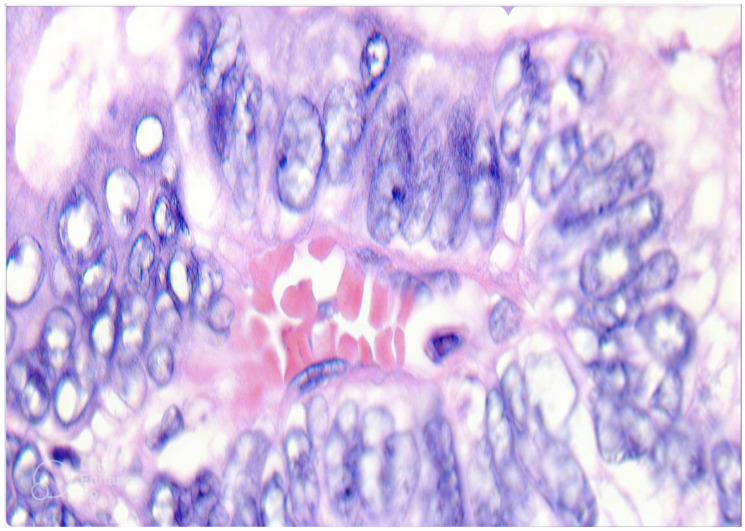
Sample of LC25000 Dataset [43].

LC25000 includes 25,000 histopathological images of lung and colon tissues, divided into five classes: colon adenocarcinoma, colon benign tissue, lung adenocarcinoma, lung benign tissue, and lung squamous cell carcinoma (a sample is shown in Figure 5). It supports both binary (normal vs. cancerous) and multi-class classification, and is particularly useful for evaluating model generalization across multiple organs and cancer types. Each class contains an equal number of images (5000), making it well suited for baseline evaluation and cross-dataset testing.

A comparative overview of the characteristics of the dataset, including organ type, magnification, number of classes, and annotation types, is presented in Table 1. Also, it should be noted that the datasets consists of image-level histopathological samples rather than enrolled patient cohorts; therefore, the study reports classification results based on labeled images categorized as benign or malignant subtypes, without subject-level or borderline tumor annotations.

#### 2.1.2. Data Augmentation Using DCGAN

To address class imbalance, especially in the BreakHis dataset’s malignant subtypes, a deep convolutional generative adversarial network (DCGAN) was employed to synthetically generate minority-class images [24,25,26].

The DCGAN architecture comprises the following:

Generator: A stack of transposed convolutional layers that progressively upscales a random noise vector into a synthetic histopathology image, following standard DCGAN design principles widely adopted in medical image synthesis [24].

The discriminator is implemented as a convolutional neural network trained to distinguish real images from synthetically generated samples and optimized using binary cross-entropy loss. Training of the discriminator–generator framework was carried out for 5 epochs using the Adam optimizer with a (learning rate of 2 × 10^−4^), and a batch size of 64. After training, the generator was sampled to produce up to 500 synthetic images for each underrepresented class. All generated samples were manually reviewed to remove unrealistic artifacts before being incorporated into the training dataset. The inclusion of these high-quality synthetic images enhanced the robustness of the downstream classification models and effectively mitigated overfitting, particularly in multiclass classification scenarios.

To mitigate class imbalance, DCGAN-based data augmentation was applied only to the training set. For the BreakHis dataset (multi-class classification), the original number of images per class was as follows: Adenosis: 113 images, Ductal carcinoma: 903 images, Fibroadenoma: 260 images, Lobular carcinoma: 170 images, Mucinous carcinoma: 222 images, Papillary carcinoma: 142 images, Phyllodes tumor: 121 images, Tubular adenoma: 150 images. Each minority class was augmented to obtain a balanced number of training samples. After augmentation, we had the following numbers of images per class in the training set: Adenosis: 787 images, Fibroadenoma: 640 images, Lobular carcinoma: 730 images, Mucinous carcinoma: 678 images, Papillary carcinoma: 758 images, Phyllodes tumor: 779 images, Tubular adenoma: 750 images. The ductal carcinoma class did not require augmentation due to it having a sufficient number of original samples.

To address class imbalance and enhance the diversity of training samples, the DCGAN was trained exclusively on the training subset, and synthetic images were generated only for underrepresented classes to achieve a more balanced class distribution. No synthetic samples were introduced into the validation or test sets to avoid any form of information leakage. The impact of DCGAN-based augmentation was evaluated through an ablation study by comparing model performance trained on the original dataset alone against models trained with additional DCGAN-generated samples. Experimental results show that incorporating DCGAN augmentation consistently improved classification performance across both binary and multi-class tasks, particularly in terms of F1-score and overall accuracy. This confirms that the observed performance gains are attributable to improved data diversity rather than overfitting or data leakage.

#### 2.1.3. Deep Learning Models

This study employs several state-of-the-art deep learning architectures to perform binary and multiclass classification of histopathological images. The models include InceptionV3, EfficientNetB0, and the Vision Transformer (ViT), selected for their established effectiveness in medical image analysis tasks as shown in Table 2. Each model was fine-tuned using transfer learning to adapt pretrained weights to the specific characteristics of the target datasets.

InceptionV3: A convolutional neural network composed of inception modules that perform multi-scale feature extraction, optimizing computational efficiency while maintaining accuracy.

EfficientNetB0: A lightweight CNN obtained through neural architecture search. It applies compound scaling to balance the depth, width and resolution of the network, making it suitable for deployment in resource-limited settings.

Vision Transformer (ViT): A transformer-based model that segments input images into patches and applies self-attention mechanisms to learn global dependencies. ViT demonstrated strong intra-dataset classification performance, particularly on high-resolution datasets.

All models were trained using consistent pre-processing, dataset splitting (70:15:15), and optimization protocols to ensure a fair comparison. Binary cross-entropy or categorical cross-entropy was used depending on the classification task. The model architecture was extended with a fully connected classification head customized to the number of output classes.

Additionally, a deep convolutional generative adversarial network (DCGAN) was integrated for synthetic data augmentation. By generating high-fidelity samples for underrepresented classes, DCGAN helped reduce class imbalance and improve model generalization across binary and multiclass tasks.

#### 2.1.4. Evaluation Strategy

To ensure fair and consistent evaluation across all models and datasets, a unified evaluation protocol was used. Each dataset was randomly split into training (70%), validation (15%), and testing (15%) sets.

Model performance was assessed using a comprehensive set of classification metrics, including accuracy, precision, recall, F1-score, and confusion matrix. For binary classification tasks, the Receiver Operating Characteristic Area Under the Curve (ROC-AUC) was also calculated to evaluate the model’s ability to distinguish between classes, particularly in imbalanced settings.

To mitigate overfitting, early stopping was applied based on validation loss with a defined patience threshold. Learning rate schedulers were also employed to dynamically adjust training rates for improved convergence. The best model checkpoint, determined by the highest validation accuracy or the lowest validation loss, was used for the final testing.

The generalizability of the model was evaluated by evaluating the performance across different datasets (BreakHis, LC25000, BACH) and varying magnification levels (e.g., 40×, 100×). This cross-dataset evaluation provided insight into the robustness and transferability of each model architecture.

#### 2.1.5. Web/Mobile Application for Real-Time Inference

**Figure 6:**
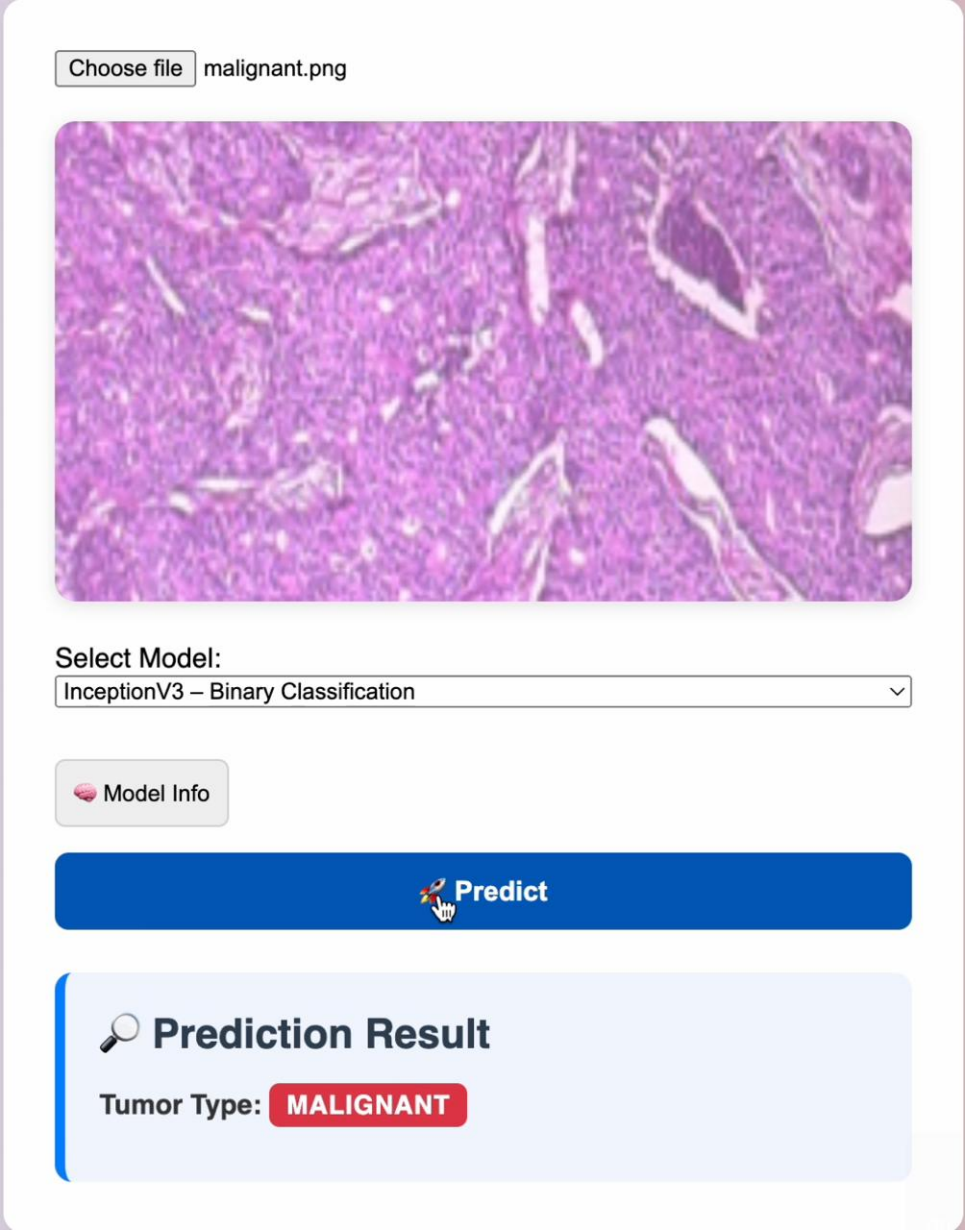
Sample screenshot of the developed web application showing the cancer-type prediction interface.

To enhance the practical usability of the proposed framework, a web/mobile application was developed that enables real-time cancer type prediction directly from histopathological images, as shown in Figure 6. The application provides an intuitive interface through which users can upload or capture an image of a tissue sample. Once the image is selected, the user chooses from the available trained deep learning models (InceptionV3, EfficientNetB0, or ViT). The selected model is executed on a remote inference server to ensure fast and resource-efficient processing.

After the model processes the input, the app displays the predicted cancer class along with confidence scores, offering immediate diagnostic support. This integration bridges the gap between research and applied clinical usage by providing a lightweight, accessible, and scalable tool for pathologists, medical students, and healthcare practitioners. The web/mobile deployment demonstrates the framework’s potential for real-world implementation, especially in settings where access to high-end computational resources is limited.

### 2.2. Pipeline B: Whispering Gallery Mode (WGM) Optical Sensing Framework

**Figure 7:**
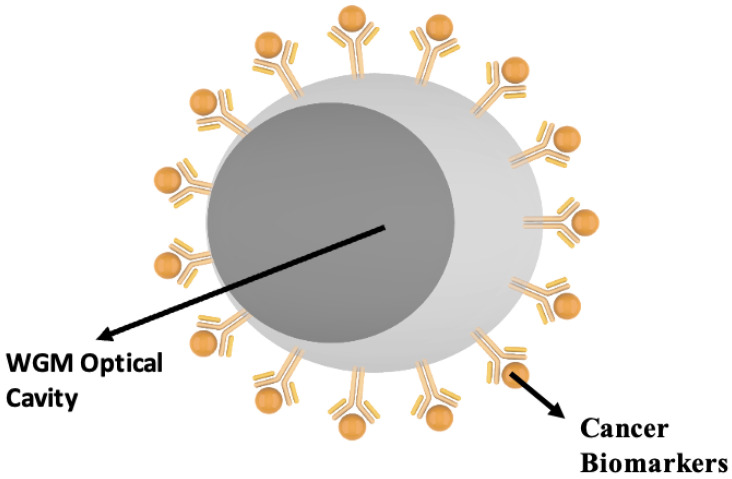
Functionalized WGM cavity for cancer biomarker detection. The figure illustrates a WGM optical sensor functionalized to selectively capture specific cancer biomarkers. Binding of target molecules, such as proteins, antigens, or nucleic acid fragments, occurs at the sensor surface.

A future-ready diagnostic framework requires the seamless integration of multimodal information to overcome the inherent limitations of morphology-only assessment. To this end, we propose a hybrid system that combines advanced deep learning algorithms for histopathological image analysis with high-sensitivity Whispering Gallery Mode (WGM) optical sensors for molecular detection. By uniting morphological and molecular-level data, this approach addresses the diagnostic uncertainty that arises when tissue features alone are insufficient for a definitive classification, ultimately enhancing the reliability and early detection capability of the system. Whispering Gallery Mode sensors offer a particularly powerful platform for label-free chemical and biomedical detection, with significant potential in early-stage cancer diagnosis. These sensors operate on the principle that WGM cavities can be functionalized to selectively capture specific cancer biomarkers as shown in Figure 7. Binding of molecules such as proteins, antigens, or nucleic acid fragments to the sensor surface alters the local refractive index. Such changes produce detectable shifts in the resonant wavelength or frequency of the cavity, allowing for highly sensitive and quantitative molecular detection [44]. Integrating this molecular sensing capability with AI-driven histopathology establishes a robust, multimodal analytical framework that bridges the gap between cellular morphology and molecular pathology.

**Figure 8:**
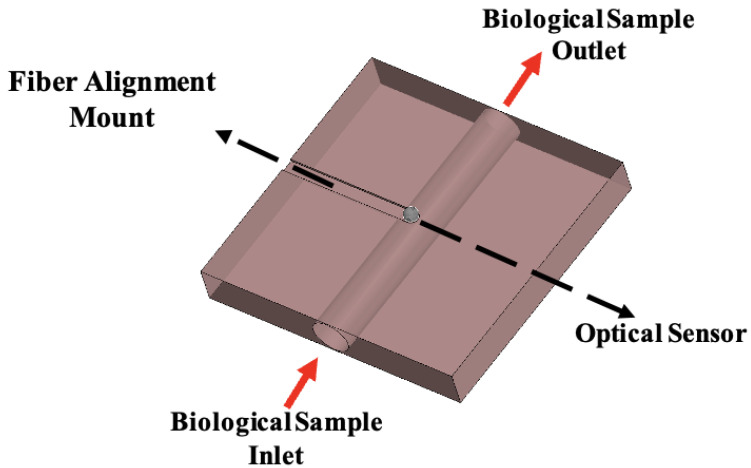
Microfluidic–optical interface enabling WGM-based biochemical sensing. The figure illustrates the microfluidic platform that guides the biological sample from the inlet to the outlet while directing it across the optical sensing region.

The precision routing machine plays a critical role in the development of WGM-based sensor platforms by enabling the fabrication of precisely controlled microfluidic channels and integrated optical interfaces. It facilitates the creation of microfluidic pathways that deliver fluids with regulated flow across the sensing region, alongside precision cutouts, grooves, and fiber alignment mounts that ensure stable, low-loss optical coupling. These microfabrication capabilities, initially employed for the delivery of biopsy-derived fluids to WGM sensing modules, are readily extendable to other polymeric WGM-based sensors, including olfactory detection systems for humanoid robots. By integrating these fluidic and optical components into a single, customizable platform, the routing process ensures reliable sample transport, reproducible sensor performance, and robust optical coupling—features that are essential for the accurate detection of WGM spectral shifts. This fabrication process directly supports the microfluidic–optical interface illustrated in Figure 8, where biological samples flow from inlet to outlet while interacting with the WGM sensing region. For the polymeric olfactory sensor, the routed PDMS cavities and integrated optical interfaces enable odorants to interact consistently with the resonator, resulting in measurable changes in the WGM transmission spectrum [45]. The polydimethylsiloxane (PDMS) forms the primary sensing cavity, which is sealed with a permeable PDMS membrane that selectively allows odorants to reach the resonator while mitigating environmental interference. Membrane thickness was systematically varied to optimize sensor sensitivity, and WGM wavelength shifts were quantified using a cross-correlation tracking algorithm. This configuration supports real-time, portable detection, effectively functioning as an olfactory prosthetic. Consequently, the same high-resolution micromachining principles that facilitate controlled biological fluid delivery can be applied to the fabrication of polymeric WGM sensors, enhancing their reliability, sensitivity, and portability. This demonstrates the versatility of the routing approach for diverse applications in both biomedical and robotic sensing domains.

**Figure 9:**
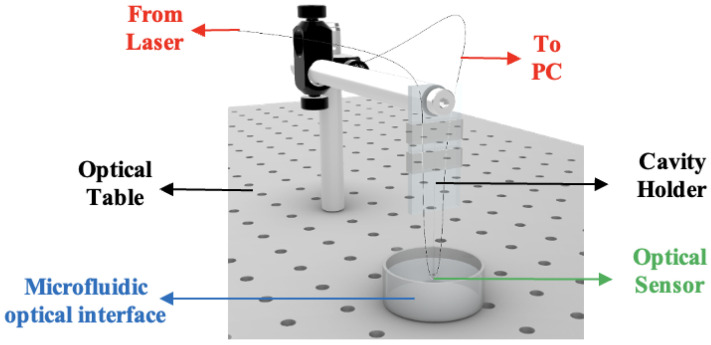
Optical sensor setup with the microfluidic chip. The diagram illustrates the integration of the Whispering Gallery Mode (WGM) optical sensor with a precision-fabricated microfluidic chip, showing controlled fluid delivery from biopsy samples across the sensing region and the optical interfaces that ensure stable, low-loss coupling for robust molecular detection.

The integrated experimental setup used for the Whispering Gallery Mode (WGM) optical sensing measurements with the microfluidic chip is shown in Figure 9. In this configuration, a laser source is coupled through the optical table into the cavity holder, where the WGM resonator is precisely aligned for stable light confinement. The microfluidic optical interface delivers controlled fluid samples, such as biopsy-derived analytes, directly across the sensing region, ensuring consistent and low-loss interaction between the guided light and the flowing medium. The resulting beam deflection functions as an external compression force on the isolated microsphere, producing measurable WGM resonance shifts. The arrangement enables real-time interrogation of molecular changes within the microfluidic environment, providing a robust platform for high-sensitivity detection and analysis.

As described earlier, the sample flows through both Pipeline (A) and Pipeline (B) to generate complementary diagnostic outputs.

## 3. Results

The results presented in this section correspond to Pipeline A of the proposed hybrid diagnostic framework, which focuses on histopathological image analysis using deep learning models. Binary and multiclass classification tasks were performed on the BreakHis, BACH, and LC25000 datasets to evaluate the performance of InceptionV3, EfficientNetB0, MobileNetV2, and Vision Transformer (ViT) architectures, both with and without DCGAN-based data augmentation. Performance metrics, including accuracy, precision, recall, F1-score, confusion matrices, and ROC curves, were used to quantify the effectiveness and reliability of the models. Results for Pipeline B (WGM optical sensing) will be explored in future work, while the current evaluation provides a detailed assessment of the AI-driven histopathology component of the framework.

**Figure 10:**
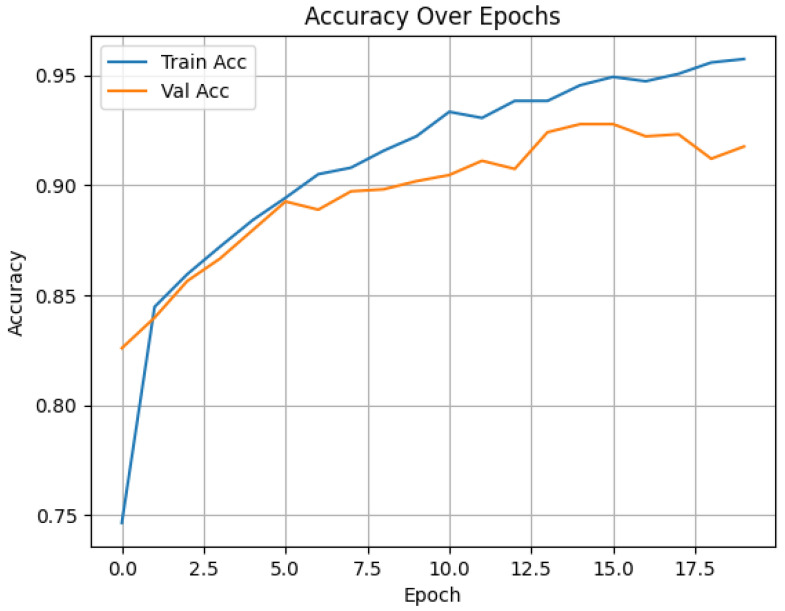
Accuracy curve (Inception V3 & DCGAN & BreakHis).

The evaluation involved binary and multiclass classification tasks using EfficientNetB0, InceptionV3, MobileNetV2, and Vision Transformer (ViT) models.The training and validation accuracy curves show consistent improvement over 20 epochs, with the training accuracy reaching 96% and the validation accuracy stabilizing around 93%. This indicates effective learning and good generalization without significant overfitting, as shown in Figure 10.

**Figure 11:**
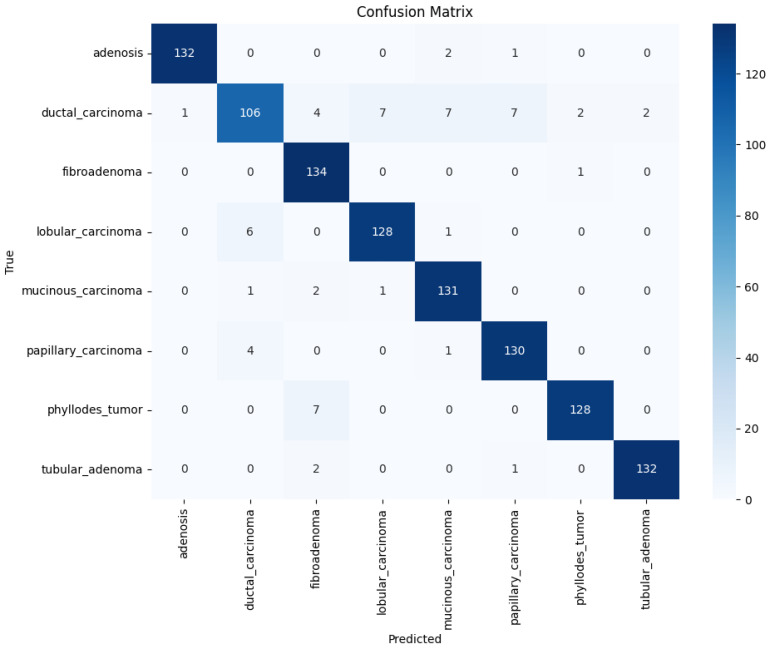
Confusion Matrix (Inception V3 & DCGAN & BreakHis).

The confusion matrix highlights strong classification performance across all eight tumor subtypes, with high true positive rates. Minor misclassifications occurred mainly between histologically similar classes such as ductal and lobular carcinoma. It should be noted that the BreakHis dataset does not provide borderline or malignant annotations for phyllodes tumors; phyllodes cases are included exclusively under the benign category. Consequently, the multiclass classification shown in Figure 11 reflects the eight BreakHis subtypes rather than a benign–borderline–malignant phyllodes stratification.

Figure 11 presents the confusion matrix for the InceptionV3 model trained on the BreakHis dataset with DCGAN augmentation, illustrating the improved classification consistency between classes.

**Figure 12:**
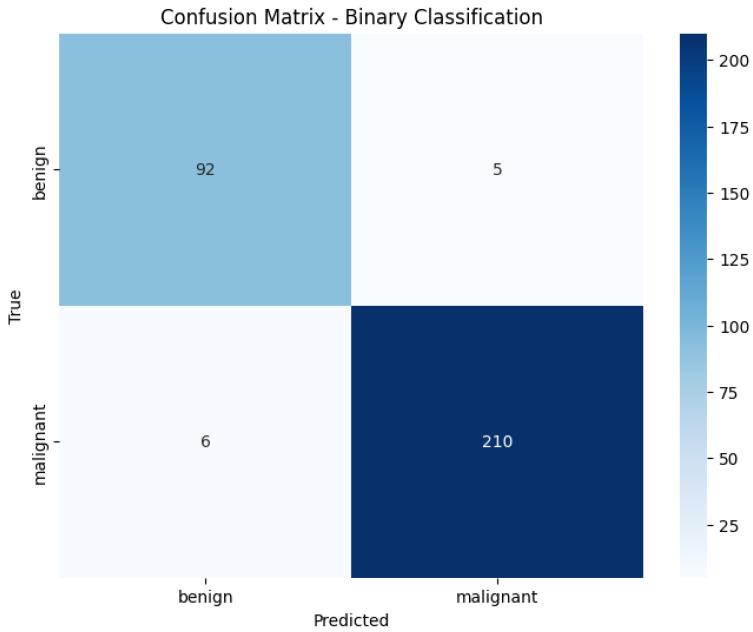
Confusion Matrix (InceptionV3 on BreakHis (Binary)).

The confusion matrix in Figure 12 illustrates the strong performance of the InceptionV3 model in distinguishing between benign and malignant tumors in the BreakHis binary dataset. Of 313 test samples, 302 were correctly classified, with only 11 misclassifications. The model demonstrates high sensitivity (recall for malignant) and specificity (recall for benign), indicating its reliability in clinical screening scenarios.

**Figure 13:**
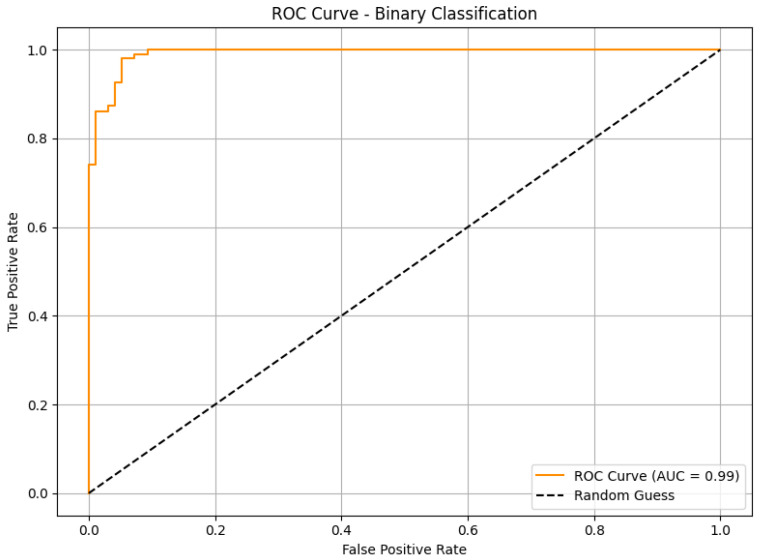
ROC Curve (InceptionV3 on BreakHis (Binary)).

The ROC curve in Figure 13 shows the model’s ability to discriminate between benign and malignant classes. The Area Under the Curve (AUC) is nearly 1.0, which signifies excellent classification performance. This high AUC validates the robustness of the model and its ability to make confident predictions even under class imbalance.

**Figure 14:**
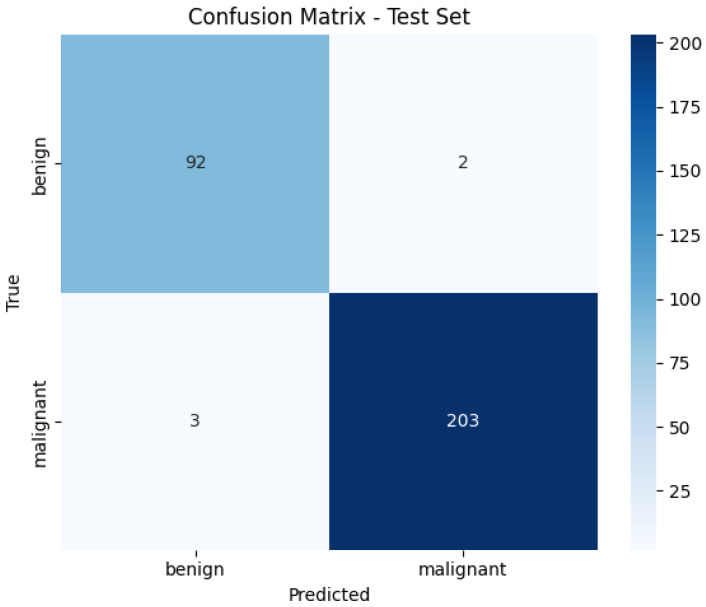
Confusion Matrix of ViT on BreakHis Dataset.

Figure 14 illustrates the confusion matrix for the Vision Transformer (ViT) model in the binary classification task using the BreakHis dataset. The model correctly classified 92 benign and 203 malignant cases, with only five misclassifications in total. These results demonstrate the strong discriminative ability of the model in separating benign and malignant samples, achieving high sensitivity and specificity. The low false-positive and false-negative rates reflect the robustness of the transformer architecture for histopathological image classification.

**Figure 15:**
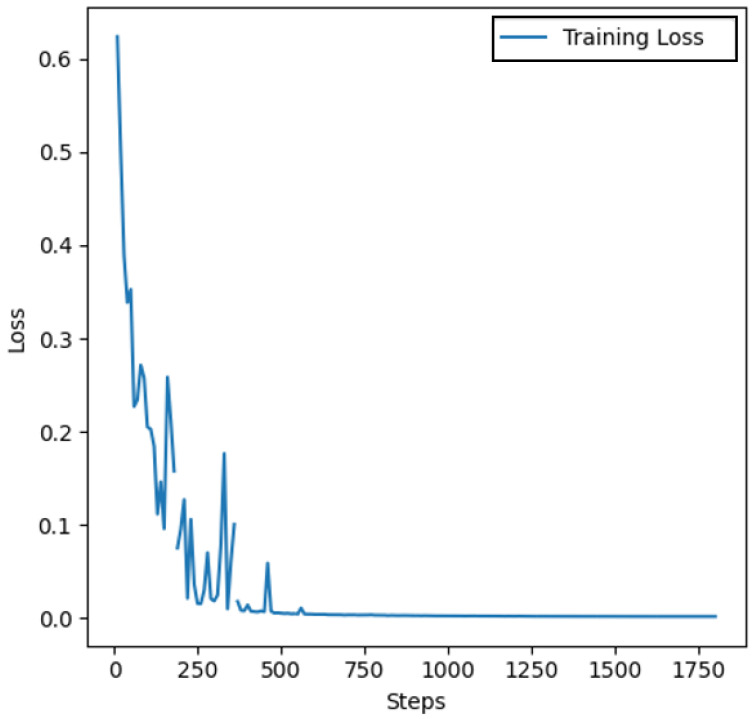
Training loss accuracy curves of the Vision Transformer (ViT) on the BreakHis dataset. The x-axis represents training steps recorded at the batch level rather than epoch indices, reflecting the batch-wise logging strategy used during model training.

Figure 15 presents the training loss and validation accuracy curves of the ViT model during training on the BreakHis dataset. The loss curve shows a rapid decrease in both training and validation loss during the initial epochs, followed by smooth convergence, indicating effective learning and minimal overfitting. The validation accuracy remains consistently high, approaching 99%, confirming that the model generalizes well on unseen data. These patterns validate the suitability of ViT for biomedical image analysis when sufficient data augmentation and regularization are applied.

Table 3 summarizes the performance of all the models evaluated across different datasets using key classification metrics.

To ensure clinical relevance and diagnostic reliability, the model outputs were reviewed and validated by a certified pathologist specializing in oncologic diagnostics at a major cancer center in the United States. The specialist confirmed that the proposed system successfully distinguished between benign and malignant cases, as well as across multiple tumor subtypes. The pathologist recommended integrating a segmentation module to highlight tumor regions, improving clarity and visual interpretability during diagnostic assessment. Secondly, they recommended extending the system to support multi-organ cancer classification to broaden its clinical applicability.

As noted, results for Pipeline B (WGM optical sensing) are not reported in the current study and will be explored in future work, whereas the present manuscript provides a detailed evaluation of the AI-driven histopathology component (Pipeline A) of the framework.

## 4. Discussion

The results presented in this study demonstrate the effectiveness of deep learning models, particularly InceptionV3 and Vision Transformer (ViT), for binary and multiclass classification of histopathological images. The integration of DCGAN-based augmentation substantially improved model performance, especially for underrepresented malignant classes in the BreakHis dataset. ViT achieved the highest accuracy for intra-dataset evaluation (98.82%), highlighting the potential of transformer-based architectures in capturing global contextual information in high-resolution histopathology images.

Compared to previous studies, our framework provides competitive performance while maintaining robustness across multiple datasets, as shown in Table 4. For instance, while ViT reached 98.82% accuracy in binary classification on BreakHis, related work reported slightly higher accuracy (99.89%) [7]. However, in multiclass classification, InceptionV3 with DCGAN augmentation outperformed previous methods (94.45% vs. 82.2%) [46], demonstrating the advantage of combining generative augmentation with conventional CNN architectures.

The confusion matrices reveal that misclassifications occurred primarily between histologically similar tumor subtypes, such as ductal and lobular carcinoma, which aligns with the challenges reported in histopathological literature [3]. These results emphasize the importance of incorporating additional modalities or features, such as segmentation maps or molecular biomarkers, to further enhance diagnostic precision.

Despite these strengths, some limitations remain, as cross-dataset evaluation revealed reduced performance (e.g., ViT on LC25000 achieved 57% accuracy), indicating challenges in model generalization across different organs and tissue types. At the same time, the findings highlight the potential of deep learning-based histopathology classification systems for clinical application, particularly when enhanced with generative augmentation and multimodal integration. Future work should focus on leveraging multi-organ datasets, transfer learning techniques, and domain adaptation strategies to enhance generalization, while extending the framework to include semantic segmentation, additional data modalities, and multi-organ cancer classification to further improve diagnostic accuracy and clinical relevance.

Moreover, the integration of a web/mobile application illustrates the translational potential of this framework, enabling real-time inference and practical usage by pathologists. By providing both confidence scores and visual outputs, the system supports informed clinical decision-making while reducing workload and minimizing human error.

From a broader perspective, the proposed hybrid methodology, which integrates deep-learning-based morphological analysis with machine-learning-based Whispering Gallery Mode (WGM) optical sensing, represents a promising future direction. By combining structural and biochemical information, this approach enables automated extraction and interpretation of complex datasets, offering a multimodal diagnostic pipeline that is more robust, sensitive, and clinically reliable than image-only approaches.

## 5. Conclusions

This study presents a Hybrid Multimodal Diagnostic Framework that combines deep-learning-based histopathological image analysis with machine-learning-based optical sensing using Whispering-Gallery Mode (WGM) principles. By integrating morphological image features with ultra-sensitive molecular detection, this novel hybrid approach enables a more comprehensive assessment of tissue abnormalities, enhancing diagnostic confidence and supporting the resolution of ambiguous or challenging cases.

The histopathological imaging framework was evaluated using the BreakHis, BACH, and LC25000 datasets, encompassing diverse cancer types and magnification levels. Multiple deep learning architectures, including InceptionV3, EfficientNetB0, and Vision Transformer (ViT), were implemented and augmented with DCGAN-based data synthesis to address class imbalance and improve generalization. EfficientNetB0 achieved strong performance in binary classification tasks, while ViT showed notable accuracy in intra-dataset evaluations. Specifically, InceptionV3 with DCGAN augmentation achieved a multi-class accuracy of 94.45% and binary classification accuracy of 96.49% on BreakHis; ViT reached 98.82% on BreakHis and 57% on LC25000; and EfficientNetB0 achieved 67.67% accuracy. These results demonstrate the complementary strengths of convolutional and transformer-based models across different datasets and tasks.

A real-time web-based interface was also developed to facilitate practical clinical deployment, enabling rapid and reliable predictions. This interface bridges the gap between computational pathology research and routine clinical workflows, with the potential to reduce diagnostic errors, accelerate pathology processes, and provide enhanced decision support, particularly in low-resource healthcare environments.

Future work includes the integration of semantic segmentation models to precisely delineate tumor regions, improving interpretability and enabling more targeted diagnostics. Incorporating additional clinical data, such as radiological imaging, laboratory tests, and patient history, can further enhance system robustness and generalizability. Moreover, expanding the framework to multi-organ cancer classification would increase its clinical applicability across diverse tissue types. In addition, probabilistic models such as Bayesian Networks will be explored to complement deep learning approaches by providing interpretable, uncertainty-aware decision support for complex, multidimensional clinical data. Furthermore, the evaluation of Pipeline B (WGM optical sensing) will be conducted to assess its contribution to diagnostic accuracy and its integration with the AI-driven histopathology component, providing a more comprehensive multimodal framework. We also plan to investigate advanced fusion strategies, including feature-level attention mechanisms, to adaptively emphasize the most informative modality-specific features and maximize complementary contributions from histopathological and WGM-derived data. Finally, we plan to assess the computational efficiency of the system under real-time operating conditions, including inference time per sample, hardware specifications, and runtime performance, to evaluate the practicality and feasibility of deploying the framework in clinical settings.

In summary, the combination of convolutional and transformer-based deep learning models with machine-learning-enhanced WGM multimodal sensing offers a scalable, clinically relevant, and high-performing platform for automated histopathological cancer detection.

## Figures and Tables

**Table 1:** Comparison of Commonly Used Histopathological Datasets.

Dataset	Organ	Number of Classes	Annotation Type
BACH [42]	Breast	4 (Normal, Benign, In Situ, Invasive)	Region-level segmentation and image-level labels
BreakHis [41]	Breast	8 (4 benign, 4 malignant subtypes)	Image-level class labels only; magnification-specific
LC25000 [43]	Lung, Colon	5 (e.g., benign, malignant, normal)	Image-level class labels only; useful for binary and multiclass classification

**Table 2:** Summary of model architectures used in this study.

Model	Architecture	Attention Mechanism	Input Size	Output
Inception V3	Inception V3 CNN	None	299×299×3	Global Average Pooling + Fully Connected Softmax
EfficientNet-B0	EfficientNet-B0 CNN	Squeeze-and-Excitation (SE) blocks	224×224×3	Global Pooling + Fully Connected Softmax
Vision Transformer (ViT)	Transformer Encoder	Multi-Head Self-Attention (MHSA)	224×224×3 (patch-based)	Classification token + Fully Connected Layer

**Table 3:** Performance Comparison Across Models and Datasets.

Model	Dataset	Accuracy (%)	Precision	Recall	F1-Score
EfficientNetB0	BreakHis	67.67	0.68	0.98	0.80
ViT	BreakHis	98.82	0.98	0.98	0.98
ViT	LC25000 (test)	57.0	0.59	0.57	0.57
InceptionV3	BreakHis (Binary)	96.49	0.96	0.96	0.96
InceptionV3	BreakHis (Multi-class)	94.45	0.94	0.94	0.94

**Table 4:** Performance Comparison with Related Work.

Model	Dataset	Accuracy (Our Work)	Accuracy (Related Work)
ViT [7]	BreakHis (Binary Classification)	98.82%	99.89%
Inception V3 [5]	BreakHis (Binary Classification)	98.82%	99.89%
Inception V3 [46]	BreakHis (Multi Classification)	94.45%	82.2%

## Data Availability

The data supporting the findings of this study are publicly available. The histopathological image datasets used in this work include the BreakHis dataset, the BACH dataset, and the LC25000 dataset, all of which are available on Kaggle.
